# Crazing Effect on the Bio-Based Conducting Polymer Film

**DOI:** 10.3390/polym13193425

**Published:** 2021-10-06

**Authors:** Pei-Yi Wong, Akiyoshi Takeno, Shinya Takahashi, Sook-Wai Phang, Azizah Baharum

**Affiliations:** 1 Department of Chemical Sciences, Faculty of Science and Technology, Universiti Kebangsaan Malaysia, Bangi 43600, Selangor, Malaysia; wvyerwong@gmail.com; 2 Department of Chemistry and Biomolecular Science, Faculty of Engineering, Gifu University, Gifu 501-1193, Japan; takeno@gifu-u.ac.jp (A.T.); shinyat@gifu-u.ac.jp (S.T.); 3 Department of Physical Science, Faculty of Applied Sciences, Tunku Abdul Rahman University College, Jalan Genting Kelang, Kuala Lumpur 53300, Malaysia; 4 Polymer Research Center, Faculty of Science and Technology, Universiti Kebangsaan Malaysia, Bangi 43600, Selangor, Malaysia

**Keywords:** crazing, biodegradation, polyaniline, polylactic acid

## Abstract

The biodegradability problem of polymer waste is one of the fatal pollutFions to the environment. Enzymes play an essential role in increasing the biodegradability of polymers. In a previous study, antistatic polymer film based on poly(lactic acid) (PLA) as a matrix and polyaniline (PAni) as a conductive filler, was prepared. To solve the problem of polymer wastes pollution, a crazing technique was applied to the prepared polymer film (PLA/PAni) to enhance the action of enzymes in the biodegradation of polymer. This research studied the biodegradation test based on crazed and non-crazed PLA/PAni films by enzymes. The presence of crazes in PLA/PAni film was evaluated using an optical microscope and scanning electron microscopy (SEM). The optical microscope displayed the crazed in the lamellae form, while the SEM image revealed microcracks in the fibrils form. Meanwhile, the tensile strength of the crazed PLA/PAni film was recorded as 19.25 MPa, which is almost comparable to the original PLA/PAni film with a tensile strength of 20.02 MPa. However, the Young modulus decreased progressively from 1113 MPa for PLA/PAni to 651 MPa for crazed PLA/PAni film, while the tensile strain increased 150% after crazing. The significant decrement in the Young modulus and increment in the tensile strain was due to the craze propagation. The entanglement was reduced and the chain mobility along the polymer chain increased, thus leading to lower resistance to deformation of the polymer chain and becoming more flexible. The presence of crazes in PLA/PAni film showed a substantial change in weight loss with increasing the time of degradation. The weight loss of crazed PLA/PAni film increased to 42%, higher than that of non-crazed PLA/PAni film with only 31%. The nucleation of crazes increases the fragmentation and depolymerization of PLA/PAni film that induced microbial attack and led to higher weight loss. In conclusion, the presence of crazes in PLA/PAni film significantly improved enzymes’ action, speeding up the polymer film’s biodegradability.

## 1. Introduction

Over the past, trillion tons of plastic packaging derived from fossil fuel were invented to fulfill the demands of society. Consequently, high tensile performance and low cost of plastic packaging contributed to a significant raise in municipal plastic wastes. In 2015, two-thirds out of 8.3 billion tons of plastic packaging accumulated and remained intact in the environment. The accumulation of plastic packaging threatens the health risks of human and marine life in the world [1]. Nevertheless, the presence of plastic packaging pollution also remarked in greenhouse gas emissions towards the lifecycle of the ecosystems. The environmental climate change issues due to fossil fuel-based plastic packaging should be a focus, and practical action is urged to be taken.

An awareness of the environmental problem, poly(lactic acid) (PLA), one of the bio-based polymers, was developed. The past study reported that PLA could be used to substitute fossil fuel-derived polymers, especially in packaging applications [2], which is due to the fact that PLA exhibited good mechanical properties, high biodegradability, and good biocompatibility. Thus, PLA played an essential role in green chemistry and reduced the carbon footprint effectively [3]. PLA degradation releases non-toxic substances, including water, gas, and biomass, as the end products [4]. Despite the degradation of PLA in such a direction, the challenges of the ingested PLA still existed, which is due to the unmanaged natural environment, which failed to provide a proper condition for the microbial degradation of PLA [5]. For instance, biodegradable PLA constricts to be degraded in seawater. Indeed, the low degradation efficiency of PLA due to unsuitable condition need to be overcome [6]. Meanwhile, PLA behaves as a non-conductive polymer that also restricts the application of PLA in electronic devices packaging.

The non-conductive challenges of PLA have sparked studies in targeted developing of conductive properties in PLA through incorporating conductive materials [7]. Polyaniline (PAni) is one of the conducting polymers widely investigated due to its good biocompatibility and conductivity. Based on the previous studies, researchers have demonstrated that the blending of PAni into the PLA with optimum proportion displayed excellent performance [8]. Thus, researchers hypothesize that the application of craze technology in PLA/PAni film would be highly desirable to promote degradation, conserving the mechanical properties and be used in electronic packaging applications.

Craze is commonly found in the ceramic industries, where it is applied to the clay pieces before being fired in a kiln for curing [9]. The craze is known as forming the microvoids or minor cracks of the materials [10]. The craze tends to transform the non-oriented glassy or semi-crystalized polymeric solid into fibrous states form in polymeric solid. Generally, the polymer crazing phenomenon is observed during the creep period when the load is applied [11]. Continuous propagation of the craze zone along the tip of the crack promotes the surface disclosure of the polymer. Large surface disclosure of the polymer allows microbial degradation and increases the polymer degradation efficiency [12]. Up to date, less quantitative data has been reported on the effect of crazing on the polymer by enzymatic degradation.

In the early work of this research, antistatic polymer film was developed by incorporating PAni into the PLA polymer film. The PLA/PAni film successfully inherited the antistatic properties compatible with the ESD standard to prevent the static charges trapped on the packaging surface to avoid static charges accidents. In this research, the craze technique was implemented to solve the biodegradation problem of PLA/PAni film. The crazed PLA/PAni film was evaluated using optical and scanning electron microscopes before and after the biodegradation test. The biodegradable property for the crazed PLA/PAni film was discussed. The effect of the crazes on the mechanical properties of PLA/PAni film was analyzed using a tensile machine. Subsequently, the results of the biodegradation rate of the crazed and non-crazed PLA/PAni films with different interval times were analyzed.

## 2. Characterization Techniques

### 2.1. Materials

The chemicals used in the synthesis of PAni, such as aniline monomer (Ani) (99%), ammonium persulfate (APS) oxidant (98%), and dioctyl sodium sulfosuccinate (AOT) dopant (96%), were purchased from Sigma-Aldrich, USA. Toluene (99.5%), used as a solvent to extract PAni precipitates, was purchased from Chemiz. Hydrochloric acid (HCl, 37%) dopant used to dissolve the APS was provided from R&M Chemicals. Tetrahydrofuran (THF) (99.8%) used as the medium to dissolve PLA was provided by R&M Chemicals. Glycerol (Gly) (99.5%) in the analytical grade was purchased from Friendemann Schmidt, Washington, USA. PLA resin with a melt flow index of 6.0 g/10 min at 210 °C and a specific gravity of 1.24, was purchased from Nature Works^®^ PLA, 2003 D, USA. Sodium azide in analytical reagent grade (AR) was supplied by Systerm. The *Proteinase K* solution from *tritirachium* album ≥ 600 units/mL was supplied by Thermo Fisher Scientific (USA). In addition, the tris hydrochloride solution (pH 8.0) with a concentration of 1 M was supplied by Solarbio. Distilled water was obtained and purified by simple distillation. All the chemicals were used without further purification unless noted.

### 2.2. Preparation of Crazed PLA/PAni Film and Non-Crazed PLA/PAni Film

Two types of PLA/PAni film were prepared, including crazed PLA/PAni film and non-crazed PLA/PAni film. First, PAni was synthesized using Ani monomer, APS as oxidant, HCl as dopant, and AOT as surfactant through chemical oxidation at 0 °C for 24 h. The solution casting method was used to prepare the PLA/PAni film. Meanwhile, glycerol acts as the plasticizer in the PLA/PAni film to improve the interaction of PLA and PAni. In order to prepare the PLA/PAni, PLA in the amount of ~6.0 g was dissolved in 100 mL of THF solution with continuous stirring at 60 °C. Then, the glycerol with the amount of ~1.8 g was added to the dissolved PLA solution. After the glycerol was mixed well with the PLA solution, the synthesized PAni was added slowly into the mixture. The mixed solution was stirred at 60 °C for 24 h. The PLA/PAni film was produced by casting the mixtures onto a glass dish and dried at room temperature overnight. The PLA/PAni film was synthesized and optimized based on the crazing process applied to the prepared PLA/PAni film.

**Figure 1:**
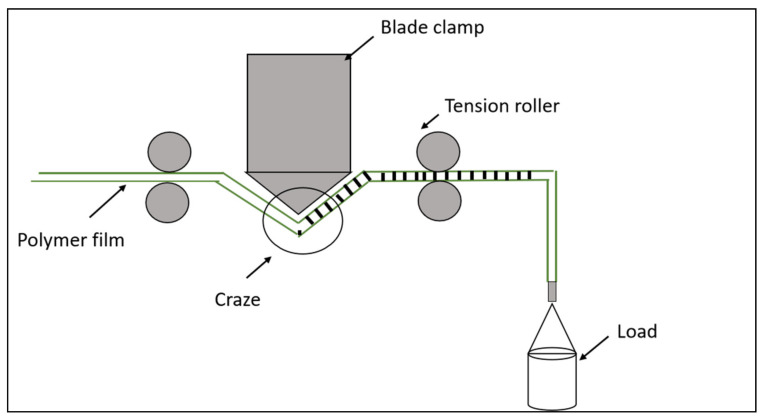
Instrument set-up used for the crazing process for the PLA/PAni film.

Next, the strip of PLA/PAni film was cut into a rectangular shape with a dimension of 70 mm (length) × 10 mm (width) and a thickness of 0.090 mm. Then, the PLA/PAni strip was clamped at the edges of the custom-made drawing device, as shown in Figure 1 [13]. The devices are associated with two film roll-up rollers, a bending blade, and stress control devices. The PLA/PAni strip was allocated in the position, as shown in Figure 1. Crazing stress applied on the strips was 6 MPa with 80° of bending angles. The PLA/PAni strip was in contact with the bending blade and created tension to form the crazes on the strip. The crazing process was conducted at room temperature with a processing rate of 20 mm/min. The presence of the crazes on the strip was identified by the annealing process. The crazed PLA/PAni strip was placed in an incubator at 60 °C for 30 min for the craze’s healing process. The strip was then cold at room temperature before the length of the strip was measured. The strip was observed under an optical microscope before and after the crazing process to confirm the formation of crazes.

### 2.3. Characterization Techniques

The crazes formation on the PLA/PAni strip was observed using Nikon optical microscopes MM-400 equipped with a camera. The surface of the PLA/PAni strip before and after the crazing process was captured. The mechanical test for crazed PLA/PAni and non-crazed PLA/PAni was performed using the EZ-L Shimadzu Tensile Tester equipped with a load cell of 1 kN. The crazed PLA/PAni and non-crazed PLA/PAni were analyzed in the condition at 23 ± 2 °C and relative humidity (RH) of 50 ± 5%. The initial gauge separation and crosshead speed were set as 15 mm and 0.5 mm/s, respectively. The mechanical properties of the crazed PLA/PAni and non-crazed PLA/PAni were recorded accordingly. The morphology images of the crazed PLA/PAni and non-crazed PLA/PAni before and after the biodegradation test was observed using scanning electron microscopy, SEM (Hitachi HiTechnologies, model SEM-4800) with accelerating voltage of 1.0 kV. The crazed PLA/PAni and non-crazed PLA/PAni were coated with platinum using a sputtering coater before being observed under SEM at a magnification of 400× and 10,000×.

Furthermore, the enzymatic degradation test was conducted using Proteinase K to study the effect of the crazes towards the crazed PLA/PAni and non-crazed PLA/PAni. Enzymatic degradation was monitored based on the weight loss of PLA/PAni film at different time intervals. The initial weight of the crazed PLA/PAni strip and non-crazed PLA/PAni strip (dimension: 10 × 70 mm) was incubated at 37 ± 1 °C in the sampling tube. The crazed PLA/PAni and non-crazed PLA/PAni strips were placed in separate sampling tubes with different contents, as shown in Table 1. At regular time intervals during the biodegradation test, the strips were collected. They were washed gently with methanol followed by distilled water and dried in a glass vacuum dryer at room temperature for 3 days to obtain the dry mass of crazed PLA/PAni and non-crazed PLA/PAni after the biodegradation test.

## 3. Results and Discussion

**Figure 2:**
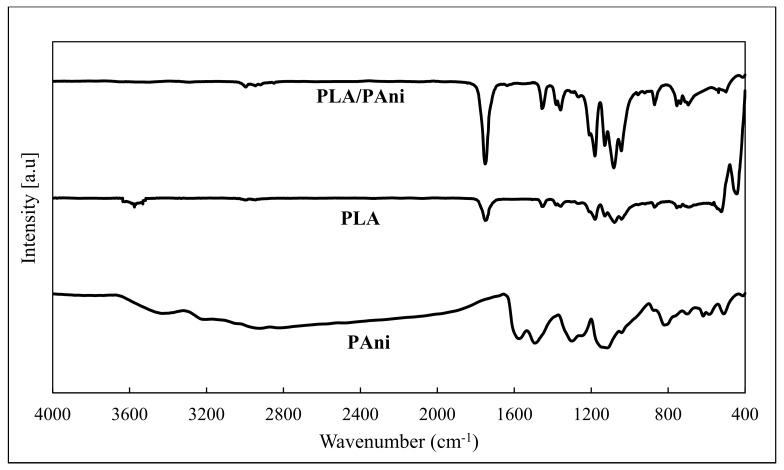
FTIR spectra of pristine PLA, pristine PAni, and PLA/PAni film.

The FTIR spectra for pristine PLA, pristine PAni, and PLA/PAni film is shown in Figure 2. Based on the FTIR spectra, the synthesized PAni revealed the characteristics of pristine PAni by giving the stretching of N−H (3215 cm^−1^), quinoid and benzenoid ring (~1400–1500 cm^−1^), C−N (1244 cm^−1^), and C−H of para-disubstituted rings of PAni (810 cm^−1^), respectively [14]. Meanwhile, the pristine PLA exhibited characteristics of C=O stretching (1746 cm^−1^). Additionally, C−H stretching (~1300–1400 cm^−1^) and C−H bending at 872 cm^−1^ indicate the characteristics of pristine PLA [15]. The PLA/PAni film was confirmed by the peaks observed at 1752 cm^−1^ (C=O), ~1400 cm^−1^ (quinoid and benzenoid ring), and ~800 cm^−1^ (C−H), respectively. All the absorption bands corresponding to the functional group of pristine PLA, pristine PAni, and PLA/PAni film were tabulated in Table 2.

**Figure 3:**
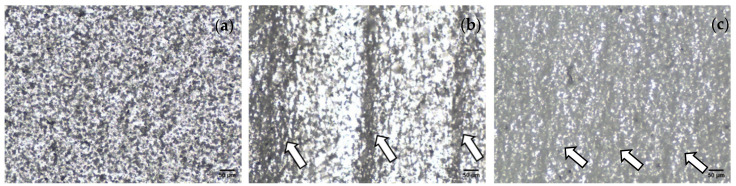
Microscope image of (**a**) non-crazed PLA/PAni film, (**b**) crazed PLA/PAni film, (**c**) crazed PLA/PAni film after the annealing process.

The optical microscopy examination on non-crazed PLA/PAni film, crazed PLA/PAni film before annealing, and crazed PLA/PAni film after annealing allows the detection and labeling of the regions attributed to the crazing process as shown in Figure 3. Figure 3a showed the PLA/PAni film without undergoing the crazing process, while Figure 3b showed the crazed PLA/PAni before the annealing treatment. Figure 3a showed the normal homogenous distribution of PLA and PAni, which is due to the fact that PAni was added slowly to the PLA and glycerol solution mixture with constant stirring to get a homogenous solution. Thus, a homogenous and consistent micrograph of PLA/PAni (Figure 3a) indicated a good dispersion of PAni in PLA [16,17].

**Figure 4:**
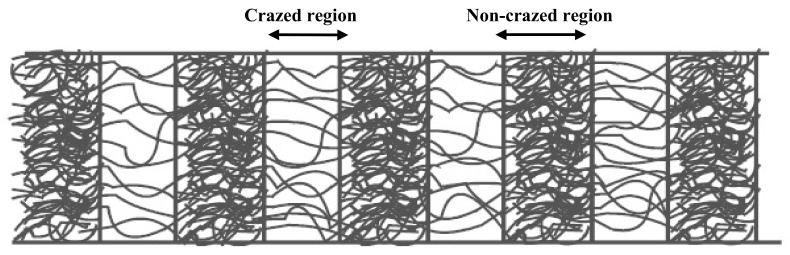
An illustration of fibrous and porous network strain that formed during the crazing process.

Figure 3b showed the lamellae lines formed in a perpendicular orientation on the crazed PLA/PAni. As indicated by the arrows in Figure 3b, the lamellae lines revealed the craze region of crazed PLA/PAni film [18]. This craze region indicated the spreading of internal stress and propagation of the craze zone along the tip of the craze. Hence, the fibrous and porous network strain formed an interval along with the PLA/PAni film and revealed in lamellae lines as shown in the optical image (Figure 3b). Thus, the crazes were successfully implemented on the PLA/PAni film after the crazing process. The fibrous and porous network strain that formed during the crazing process can be illustrated as in Figure 4.

The annealing treatment was conducted to further confirm the presence of the crazes on the PLA/PAni film. The optical image of the crazed PLA/PAni film after the annealing treatment was observed in Figure 3c. After the annealing treatment, the craze width decreased significantly compared to the crazed PLA/PAni without the annealing treatment (Figure 3b). Generally, the internal stress created during the crazing process leads to the formation of a fibrous and porous network strain, which is reflected in the lamellae line, as observed in Figure 3b. However, the fibrous and porous network strain formed during the crazing process responded to the temperature and reduced the internal stress of the polymer film during the annealing process [19]. Finally, the changes in the temperature lead to the recovery of the porous network strain in crazed PLA/PAni film and significantly decrease the number of crazes as shown in Figure 3c. Hence, the formation of crazes on the PLA/PAni film was confirmed by the annealing process.

**Figure 5:**
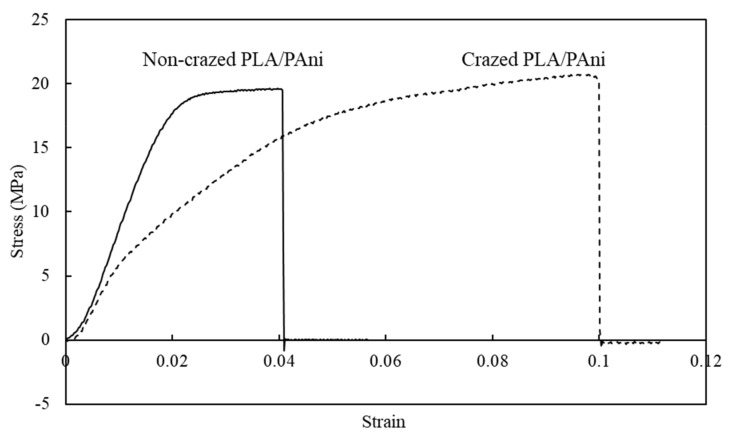
Stress–strain curve graph of non-crazed PLA/PAni film and crazed PLA/PAni film.

The mechanical properties of the crazed PLA/PAni and non-crazed PLA/PAni films are shown in Figure 5 in terms of the stress-strain curve. The curve pattern is generally almost similar but with a higher strain value for the crazed PLA/PAni film. Compared to the non-crazed film, the crazed film can reach an elongation of 150% more than the non-crazed PLA/PAni film. Generally, a higher strain rate indicates the longer time required for plastic deformation to occur [20]. The plastic deformation region of the crazed PLA/PAni film became more ductile and flexible than the non-crazed PLA/PAni film, which showed much brittle behavior. The phenomenon can be explained and supported by Figure 4, reflecting the ductile and more flexible behavior when the fibrous and porous network strain formed during the crazing process. The chain becomes easier to move and expand, hence giving more space to sustain stress. Thus, the crazed PLA/PAni film tends to have a higher elongation at break and a higher strain over time. The increasing strain for the crazed PLA/PAni film shows increases of the area under the stress-strain curve and shows a less stiffness behavior for the crazed PLA/PAni film [21]. The outcomes are in line with the result obtained in the Young modulus in this study.

From Figure 5, the non-crazed PLA/PAni film presents a higher Young’s modulus with 1113 MPa than the crazed PLA/PAni film with 651 MPa of Young’s modulus. It can be explained by the changes in the orientation of the polymer. Theoretically, the interaction between PLA and PAni created high entanglement between the polymer chain. However, the presence of crazes in the PLA/PAni film causes the mobility of polymer chains to increase. As a result, this decreases the polymer chain entanglement in the crazed PLA/PAni film and leads to lower resistance to deformation [22]. Hence, the crazed PLA/PAni exhibited less stiffness behavior and eventually decreased Young’s modulus compared to the non-crazed PLA/PAni film.

The tensile strength of crazed PLA/PAni film and non-crazed PLA/PAni film showed the resembled reading as 20.02 and 19.25 MPa, respectively. These phenomena can be explained by the structure of the polymer. Principally, the applied stress during the crazing process tends to change the orientation of the crazed polymer films rather than the backbone linkage along with the polymer [23]. Consequently, the backbone linkage for both PLA/PAni films with and without crazes remains unchanged. Thus, the amount of energy required to change the area of the film for both PLA/PAni films with and without crazes is the same. Therefore, both PLA/PAni films with and without crazes showed almost identical tensile strength reading.

**Figure 6:**
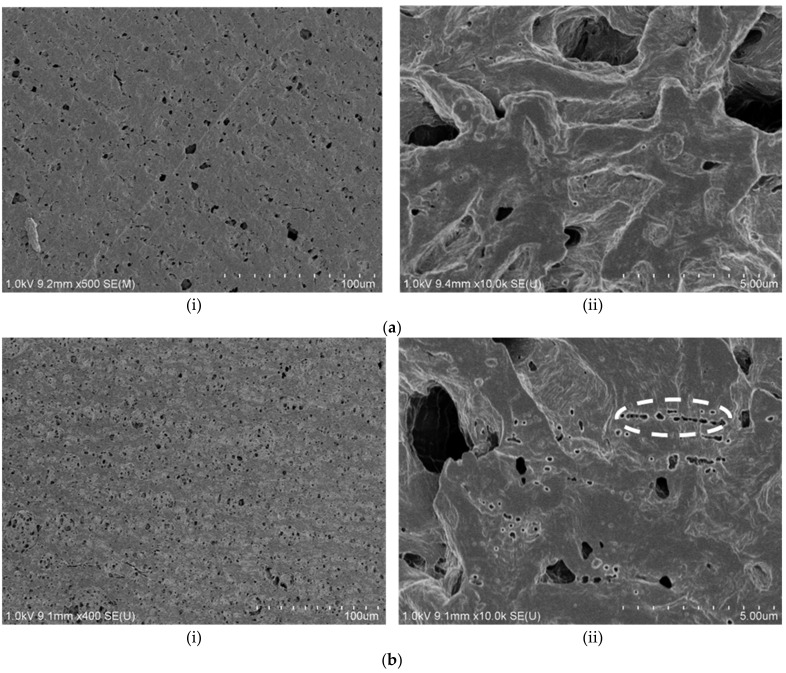
SEM morphology images of (**a**) non-crazed PLA/PAni film (i) 500× and (ii) 10,000×; (**b**) crazed PLA/PAni film (i) 400× and (ii) 10,000×.

Figure 6 shows the morphology images of non-crazed PLA/PAni film and crazed PLA/PAni film, respectively. By referring to Figure 6(ai), the PLA/PAni film without the crazing treatment showed lesser porosity and smooth surfaces. Meanwhile, the crazed PLA/PAni film in Figure 6(bi) can be observed to have a more porous structure than PLA/PAni film without the crazing treatment. This result showed the crazes-formation and reflected via the increases of porosity in the crazed PLA/PAni film morphology images. The porous structure found in the PLA/PAni film was formed by the fibrils separated by the nanosized pores as observed by the morphological images [24]. This finding is consistent with the generation of craze formation in polymer films under the action of electric discharge plasma done by Kurbanov et al. [25].

Meanwhile, by comparing Figure 6(aii,bii), it can be observed that there are diagonal cracks on the crazed PLA/PAni film. Theoretically, the crazes on the film can be identified by bright-field microscopy [26]. The diagonal cracks of the PLA/PAni film presented the craze zone, as outlined in Figure 6(bii). This eventually confirmed the craze-formation in the PLA/PAni film. The disruption of the lamellae, voiding, and fibril formation, as observed in the region of the diagonal crack, further proved the successful development of crazes in polymer film [27]. Additionally, the diagonal crack region is also recognized as a plastic deformation region where shear bands formed around the craze zone [28]. This also demonstrated that the crystalline behavior of PLA/PAni film was approaching to become an amorphous behavior [29]. As a result, the formation of crazes on the PLA/PAni film also desired to induce a higher biodegradation rate of the PLA/PAni film.

**Figure 7:**
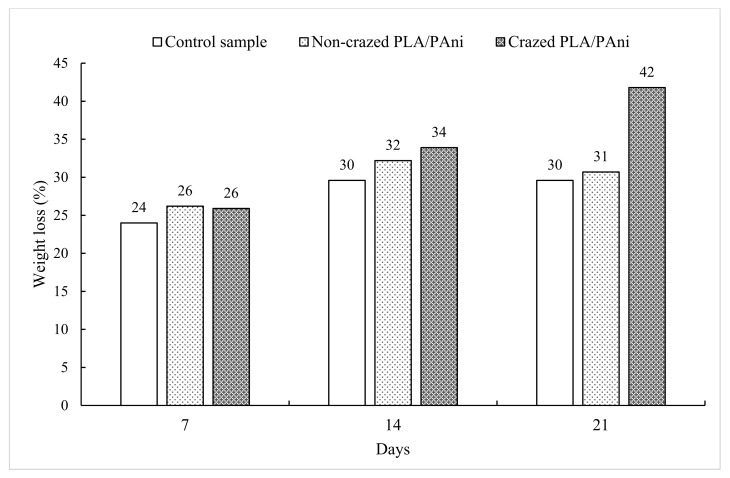
Biodegradation of non-crazed PLA/PAni and crazed PLA/PAni films at different time intervals.

This research studied the enzymatic degradation of PLA/PAni film with and without crazes within 21 days. Meanwhile, a reference film was conducted to investigate the hydrolysis of plasticizers in the PLA/PAni film. The enzymatic degradation of the films was analyzed by monitoring the weight loss of control PLA/PAni, non-crazed PLA/PAni, and crazed PLA/PAni films at different intervals of degradation time. Figure 7 showed the weight loss changes for the films under enzymatic degradation. In general, all the weight of the sample films reduced steadily by ~24% to 26% in the first 7 days. The decrease in weight of the samples for the first 7 days was due to the hydrolytic degradation of the glycerol plasticizer [30], which is attributed to the fact that glycerol played the plasticizer role in upsetting and restructuring the intermolecular polymer chain of PLA and PAni by hydrogen bonding [31]. Hydrolysis degradation of glycerol prior to happening during the degradation test as glycerol consists of hydrogen groups. Thus, the hydroxyl groups tend to have strong attraction with the hydrogen ion and form the hydrogen bonds within its structure [32]. Therefore, the weight loss in the prior days was due to the hydrolysis degradation of the glycerol in PLA/PAni film.

The weight loss of control PLA/PAni, non-crazed PLA/PAni, and crazed PLA/PAni films decrease to ~30% to 34% after 14 days of the enzymatic degradation process. At this stage, the control film reached a maximum weight loss of 30%. Thus, this indicates that the glycerol plasticizer in all the sample films had fully degraded. By comparison, the extra weight loss of 32% by the non-crazed PLA/PAni film and 34% by crazed the PLA/PAni samples showed after 14 days, which is due to the fact that the hydrolytic chain scission of ester bonds of PLA takes place after the degradation of glycerol [33]. Penetration of water into the PLA/PAni film hydrolyses at the ester group of PLA, which caused the long chains of PLA to convert into shorter chains and produce a high number of carboxyl end and hydroxyl end groups of PLA. This phenomenon resembled the explanation of Gupta and Kumar (2007) on the degradation of PLA [34].

Furthermore, the weight loss of crazed PLA/PAni film showed the highest percentages, which is 42% after 21 days of enzymatic degradation. It also found that the physical structure of the film collapsed entirely. Meanwhile, the weight loss of non-crazed PLA/PAni film showed almost linear, which is 31% after 21 days of enzymatic degradation. The highest weight reduction of 42% in crazed PLA/PAni film is correlated with the progressive deterioration of film due to the formation of crazes, which is due to the fact that crazes (in terms of pores) promote the diffusion of water in the PLA/PAni film. The high accessibility of water in PLA/PAni film caused random chain scission of the polymer chain to have occurred. Consequently, high fragmentation of polymer resulted in low molecules weight of the polymer chains [35]. Therefore, the formation of crazes accelerated the fragmentation and enhanced the depolymerization action of the polymer [36]. Hence, the crazed PLA/PAni film showed a significant decrease in the weight of the film after 21 days of degradation. This result is also in agreement with the research done by Mukhamed et al. (2020), which investigated the presence of crazes on the degradation properties of the PLA-based fibers.

**Figure 8:**
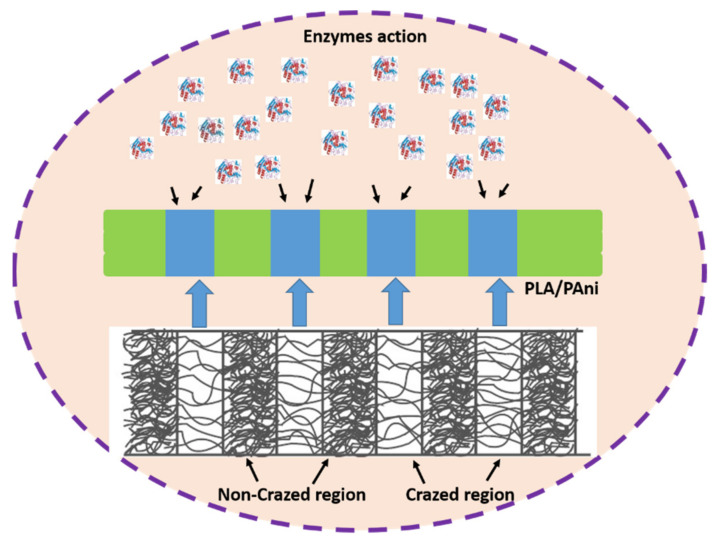
The diffusion of the enzyme in the crazed PLA/PAni film.

At the same time, the highest weight loss of crazed PLA/PAni film also contributed to the physical erosion when exposed to the biological environment [37]. Generally, the degradation process happened on the surface of the samples as enzyme molecules are too large and hardly diffuse in the PLA/PAni film [38]. However, the limitation was overcome by the formation of crazes (in terms of pores) on the PLA/PAni film. The enzymes can efficiently adsorb on the surface, diffuse into the PLA/PAni film through the pores, and accelerate the degradation of the film [39]. Meanwhile, the enzyme used in this study is Proteinase K since it showed high efficiency in the degradation of PLA, especially in contact with the water [40]. Hence, the presence of crazes in the PLA/PAni film significantly enhanced the action of enzymes and improved the biodegradability of the polymer films, which could reduce the impact of polymer on environmental pollution. The diffusion action of the enzyme into the crazed PLA/PAni film is illustrated in Figure 8.

**Figure 9:**
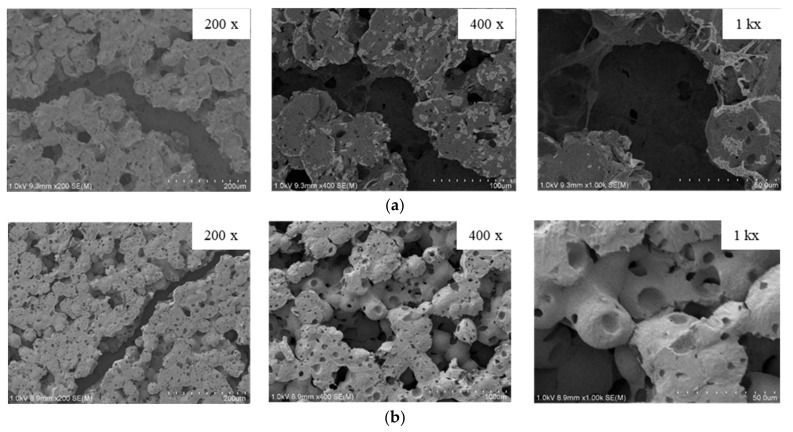
SEM morphology images of (**a**) non-crazed PLA/PAni film and (**b**) crazed PLA/PAni film after the biodegradation test.

After the biodegradation test, the morphology images of crazed and non-crazed PLA/PAni films was observed and shown in Figure 9. It is well known that PLA is an aliphatic polyester, which is susceptible to enzymatic degradation. The increase in porosity of the crazed PLA/PAni film, as shown in Figure 9b, indicates the erosion degradation of enzymes towards the polymer film. Higher porosity in the polymer film leads to a higher rate of degradation, which is aligned with the result found in Figure 7. Higher porosity in crazed PLA/PAni film promotes greater immobilized enzyme loading within the polymer film [41]. Thus, the existence of crazes in polymer film supports the assessment of enzymes for the degradation action in the polymer film. As a result, high biodegradability of crazed PLA/PAni film was produced. This result is also in line with the selective enzymatic degradation of Poly (ε-caprolactone) done by Kulkarni et al. in 2008 [42].

Meanwhile, the morphology images of non-crazed PLA/PAni film after degradation also shows a compact and firm surface in Figure 9a, while the crazed PLA/PAni film shows inflate and expand surface in Figure 9b. Principally, the enzymatic degradation is related to the chemical structure and the hydrophilic/hydrophobic nature, as well as the degree of crystallinity of the polymer. The appearance of crazes in crazed PLA/PAni film tends to increase the film’s amorphous nature. Consequently, the random hydrolytic chain scission of ester bonds in crazed PLA/PAni film allows higher diffusion of water into the amorphous region [43]. Thus, the high diffusion of water increases the degradation rate of the crazed PLA/PAni film [44]. Hence, the amorphous nature of crazed PLA/PAni film is more desired for the degradation of enzymes than the crystalline nature of non-crazed PLA/PAni film. Therefore, this also explained the highest weight loss of crazed PLA/PAni film compared to the non-crazed PLA/PAni film.

Overall, the crazed PLA/PAni film showed lamellae lines that confirmed the presence of crazes (optical microscope). At the same time, SEM morphology images reflected high porosity and diagonal cracks for the crazed PLA/PAni film, which responded to the presence of crazes in the film. On the other hand, the tensile strength is not affected due to the presence of crazes. Conversely, the flexibility of crazed PLA/PAni improved by 150%, giving a lower Young’s modulus value. Meanwhile, biodegradation of the crazed PLA/PAni film was enhanced with the presence of crazes. The summarized characterization properties of non-crazed PLA/PAni and crazed PLA/PAni films were shown in Table 3.

## 4. Conclusions

Crazing was successfully developed in the PLA/PAni film to improve the biodegradability of the polymer film. The presence of crazes in the PLA/PAni film was confirmed by optical microscope and SEM. Crazes in the PLA/PAni film did not show significant tensile strength changes but showed progressive increases in tensile strain by 150% and decreases in Young’s modulus. The non-crazed PLA/PAni film showed Young’s modulus of 1113 MPa, while the crazed PLA/PAni film showed Young’s modulus of 651 MPa. The differences that occurred in tensile strain and Young’s modulus were due to the increment in chain mobility and decrement in chain entanglement of the polymer film. Meanwhile, the crazed PLA/PAni film improved the biodegradability of the polymer film. The significant weight loss of the crazed PLA/PAni film was observed after 21 days of biodegradation as 42%. This was due to the loss of crystalline nature of the polymer film in the presence of crazes, which induced the erosion degradation of enzymes. Thus, the appearance of crazes in the PLA/PAni film enhanced the biodegradability of the polymer film. The polymer film with high biodegradability based on PLA and PAni was formulated and this study could be useful in a packaging application to lower the polymeric pollution to the environment.

## Figures and Tables

**Table 1:** Content of each sample tube during the biodegradation test.

Sample	Content of Sampling Tube
Non-crazed PLA/PAni	0.5 mg of Proteinase K and 1.0 g of sodium azide in 10 mL Tris-HCl (pH 8.0)
Control sample	1.0 g Sodium azide in 10 mL Tris-HCl (pH 8.0)
Crazed PLA/PAni	0.5 mg Proteinase K and 1.0 g sodium azide in 10 mL Tris-HCl (pH 8.0)

**Table 2:** Functional group for pristine PLA, pristine PAni, and PLA/PAni film.

Functional Group	Wavenumber (cm^−1^)		
	PLA	PAni	PLA/PAni
N−H stretching	-	3215	-
C=O stretching	1746	-	1574–1576
C=C stretching of quinoid and benzenoid	-	1584, 1487	1457, 1354
C−N stretching	-	1244	1260–1301
C−H stretching	867	810	872

**Table 3:** The summarized characterization properties of non-crazed PLA/PAni and crazed PLA/PAni films.

Properties	Non-Crazed PLA/PAni	Crazed PLA/PAni	Remarks
Optical microscope	-	Lamellae presents	Confirmed crazes presented
SEM	↓ porosity and smooth	↑ porosity & diagonal crack	Confirmed crazes presented
Tensile strength	20.02 MPa (similar)	19.25 MPa (similar)	After crazed, high tensile strength remained
Young’s modulus	1113 MPa	651 MPa	After crazed, modulus ↓, stiffness decreased
Tensile Strain	100%	250%	After crazed, 150% increment, better flexibility
Biodegradation	31%	42%	After crazed, % biodegradation ↑

## Data Availability

Not applicable.
